# Bioactive and Biodegradable Polycaprolactone-Based Nanocomposite for Bone Repair Applications

**DOI:** 10.3390/polym15173617

**Published:** 2023-08-31

**Authors:** Hosein Emadi, Mehdi Karevan, Maryam Masoudi Rad, Sorour Sadeghzade, Farnoosh Pahlevanzadeh, Mohammad Khodaei, Saber Khayatzadeh, Saeid Lotfian

**Affiliations:** 1 School of Mechanical Engineering, College of Engineering, University of Tehran, Tehran 14176-14411, Iran; 2 Department of Mechanical Engineering, Isfahan University of Technology, Isfahan 84156-83111, Iran; mkarevan@cc.iut.ac.ir; 3 Department of Chemical Engineering, Isfahan University of Technology, Isfahan 84156-83111, Iran; m.masoumirad@alumni.iut.ac.ir; 4 Shenzhen Key Laboratory of Soft Mechanics & Smart Manufacturing, Department of Mechanics and Aerospace Engineering, Southern University of Science and Technology, Shenzhen 518055, China; soroursadeghzade@gmail.com; 5 Department of Materials Engineering, Isfahan University of Technology, Isfahan 84156-83111, Iran; farnoosh.pahlevanzadeh@gmail.com; 6 Materials Engineering Group, Golpayegan College of Engineering, Isfahan University of Technology, Isfahan 84156-83111, Iran; m.khodaei@iut.ac.ir; 7 Department of Design and Mathematics, University of the West of England, Bristol BS16 1QY, UK; 8 Faculty of Engineering, University of Strathclyde, Glasgow G4 0LZ, UK

**Keywords:** baghdadite, polycaprolactone, nanocomposite, composite films, bone tissue engineering, solvent casting method, mechanical properties, thermal properties, biological properties

## Abstract

This study investigated the relationship between the structure and mechanical properties of polycaprolactone (PCL) nanocomposites reinforced with baghdadite, a newly introduced bioactive agent. The baghdadite nanoparticles were synthesised using the sol–gel method and incorporated into PCL films using the solvent casting technique. The results showed that adding baghdadite to PCL improved the nanocomposites’ tensile strength and elastic modulus, consistent with the results obtained from the prediction models of mechanical properties. The tensile strength increased from 16 to 21 MPa, and the elastic modulus enhanced from 149 to 194 MPa with fillers compared to test specimens without fillers. The thermal properties of the nanocomposites were also improved, with the degradation temperature increasing from 388 °C to 402 °C when 10% baghdadite was added to PCL. Furthermore, it was found that the nanocomposites containing baghdadite showed an apatite-like layer on their surfaces when exposed to simulated body solution (SBF) for 28 days, especially in the film containing 20% nanoparticles (PB20), which exhibited higher apatite density. The addition of baghdadite nanoparticles into pure PCL also improved the viability of MG63 cells, increasing the viability percentage on day five from 103 in PCL to 136 in PB20. Additionally, PB20 showed a favourable degradation rate in PBS solution, increasing mass loss from 2.63 to 4.08 per cent over four weeks. Overall, this study provides valuable insights into the structure–property relationships of biodegradable-bioactive nanocomposites, particularly those reinforced with new bioactive agents.

## 1. Introduction

One of medical science’s biggest challenges is eliminating bone defects [1]. Physical injuries or illness may be the root of these defects. Nevertheless, statistics show that over two million bone grafts are performed yearly, the second most common graft transplant after blood transfusion [2]. The vast majority of bone injuries, due to the regeneration ability of bone, can heal spontaneously under sufficient physiological and environmental conditions but not in significant defects due to some metabolic factors, level of defects, or unstable biomechanical properties. However, the healing process of bone defects is time-consuming. New bone generation takes place slowly because of a decreased blood supply to the fracture site and an insufficiency of calcium and phosphorus to strengthen and harden the new bone. So, bone grafts or substitute biomaterials emerge as therapeutic strategies for clinical bone surgery to fill the bone defects for reconstructing large bone segments [3,4].

The materials utilised in bone grafting are classified into three types: autografts, allografts, and xenografts. Tissue-engineered biomaterials are an alternative option that includes synthetic and biological-based substitutes [5,6]. Despite the promising results of allogeneic and autologous transplantation, the risk of disease transmission, immune incompatibility, confined availability, and donor site invalidism led to the assistance of tissue engineering to repair and regenerate damaged tissue [7]. Although the best standard clinical material for bone regeneration is autologous bone graft in osteoinduction and osteoconduction, there are concerns about donor site morbidity and the limited availability [8]. Compared with autografts, allografts have poorer integration characteristics with the host healing tissues [9]. Xenografts, in addition to the drawbacks of allografts, pose the danger of zoonotic disease transmission, and graft rejection is more probable and severe [10].

In the past decade, the urgent need for the development of clinical bone repair materials with the same results as natural bone, the engineering bone tissue, has emerged and has achieved rapid progress [11]. The bone accommodates a nanocomposite structure of polymeric collagen fibres and hydroxyapatite crystals [12]. The ideal alternative to bone should be biocompatible, repair the bone defect quickly, and not cause an adverse inflammatory response. However, it should be resorbable, osteoconductive, and osteoinductive [13]. Various materials have been developed as bone graft replacements, such as metals, ceramics, and polymers [14]. Therefore, due to the limitations of each category of material, composite and nanocomposite grafts consisting of at least two different components are developed to engineer and design grafts for targeted applications.

Nanocomposite polymers generally consist of polymers combined with inorganic/organic fillers at a nanometre scale [15]. Compared to traditional micro composites, nanocomposites exhibit improved mechanical and functional capabilities due to their interaction with the polymer matrix. Utilising nanometric manufactured structures and the high surface area-to-volume ratio that nanomaterials naturally possess, research to enhance material characteristics has steadily increased over the last two decades [16]. Because nanoparticles and polymers interact more efficiently, nanocomposite biomaterials or bio-nanocomposites offer more flexibility when designing specific properties [5,17]. Comparing them to their micro- and macro-composite counterparts, polymer nanocomposite biomaterials have better mechanical properties [18].

Among various biomaterials, biodegradable polymers offer numerous applications in the medical field, including biomedical devices, wound dressing, drug delivery and fabrication of tissue engineering, orthopaedic regeneration applications, and temporary prosthetic implants [19]. Poly(ε-caprolactone) (PCL) is a biodegradable aliphatic polyester, semi-crystalline, with a low glass transition temperature (approximately −60 °C) [20]. PCL is a rigid polymer with relatively low tensile Young’s modulus and yield stress [21].

Aliphatic polyesters such as PCL have been widely investigated for biomedical applications because of their simultaneous biodegradability and biocompatibility [22]. However, PCL has a slow degradation rate (in the range of 2–4 years) [23] and poor bioactivity, which causes fibrous tissue formation [24]. PCL is the proper material for biomedical purposes, but it faces challenges due to its low degradation rate and low mechanical properties, which have limited its application [25]. Studies reveal that adding biomaterials such as bioceramics increases the rate of bioactivity and mechanical properties [26,27]. PCL and bioactive ceramics have been evaluated by composites such as β-tricalcium phosphate [28], bioglass [29], and hydroxyapatite [30].

Studies show a promising potential for the PCL nanocomposite to be used widely in the treatment of bone fractures through grafting [31]. The PCL nanocomposite appears to play an integral part in restoring bone defects. Also, it can increase mechanical properties, the rate of degradation, and bioactivity; these are required for bone tissue engineering.

It is beneficial to fabricate thick films as a precursor to the creation of 3D scaffolds since this method makes it easier to introduce individual factors and determine how they affect cell growth. It is necessary to adequately analyse and optimise a film’s qualities before considering using it as a substrate for cell culture. In order to create dense polymer nanocomposite films, researchers have experimented with several processing methods. Nanostructures can typically be incorporated into polymers using the solution method. In this method, polymers are dissolved in an appropriate solvent containing nanoscale particles, and in the next step, the solvent is evaporated or precipitated. Solvent casting, which involves using a solvent in which the polymer is soluble, is a versatile, inexpensive, and quick method for creating polymeric nanocomposite films [32].

Nanocomposite materials based on bioactive ceramics have mainly become the most promising materials in bone defect repair due to their properties, such as excellent biocompatibility, osteoconductivity, mechanical strength, and osteogenic characteristics [33]. The matrix function holds the bioceramics together and transfers the loads applied to the bulk material. Also, bioceramics are the principal components of biocomposites that are suitable and vital for guided bone-tissue regeneration [34].

Calcium silicate ceramics have superior biological characteristics, such as bioactivity, biocompatibility, and enhanced cell interaction [35]. Among various calcium silicate ceramics, baghdadite (Ca_3_ZrSi_2_O_9_) is of significant interest mainly because of its mechanical and biological properties [36]. Furthermore, incorporating zirconium elements into the crystal structure of calcium silicates enhances its physical, mechanical, and biological properties [37]. For instance, researchers fabricated Ca_3_ZrSi_2_O_9_ scaffolds and showed an extremely novel bone formation for baghdadite scaffolds [38].

The present study developed a new biodegradable PCL-baghdadite nanocomposite using the solvent casting technique. The effect of the baghdadite addition on nanocomposites’ mechanical, thermal, biological, and surface morphology was evaluated to develop bioactive, bioresorbable, and mechanically robust nano-biocomposites. According to the authors’ literature reviews and the best of knowledge at this point in time, no studies have been reported enlightening the correlations among the fabrication, structure, and properties of a PCL-based nano-biocomposite film reinforced with baghdadite as the bio-active filler by the solvent casting method. This study was inspired by the natural structure of bone, which contains apatite nanoparticles and collagen polymer. PCL was selected as a matrix and reinforced with baghdadite nanoparticles to examine the physicochemical and biological behaviour of the specimens used in applications of bone tissue engineering.

## 2. Materials and Methods

### 2.1. Materials

Calcium nitrate tetrahydrate, ethanol, and chloroform were obtained from Merck (Darmstadt, Germany). Polycaprolactone (PCL) (ρ = 1.45 g/mL and Mn = 80,000) and zirconium (IV) oxynitrate hydrate were purchased from Sigma-Aldrich (Burlington, MA, USA), and Tetraethyl orthosilicate (TEOS) that was used as a Si source was provided by Samchun Chemical Co (Pyeongtaek-si, Republic of Korea).

### 2.2. Preparation of Baghdadite Powder

Baghdadite powder with a nanostructure was synthesised via the sol–gel process described in detail by Sadeghzade et al. [39]. To begin, initial materials were chosen, including zirconium nitrate oxide (ZrO(NO_3_)_2_), calcium nitrate tetrahydrate (Ca(NO_3_)_2_·_4_H_2_O), and tetraethyl orthosilicate (TEOS, (C_2_H_5_O)_4_Si). A mixture of TEOS, ethanol, and HNO_3_ (2 M) was created with a molar ratio of 1:8:0.16 and stirred for half an hour. Afterwards, ZrO(NO_3_)_2_ and Ca(NO_3_)_2_·4H_2_O were incorporated into the mixture, ensuring a Zr/Ca/TEOS molar ratio of 1:3:2. This solution was stirred continuously for five hours. Following this, the solution was maintained at 60 °C for a day and then dried at 100 °C over two days, which resulted in a dry gel. This dry gel was annealed at 1150 °C for 3 h as a final step.

### 2.3. Fabrication of PCL-Baghdadite Nanocomposites

The selection of the proper solvent system is the main factor in a suitable solution-mixing process. The polymer solution’s viscosity and solvent conductivity depends on the solvent density and polymer molecular weight [40]. The PCL solution with 10 *v*/*v*% chloroform was prepared and stirred for 24 h to develop a homogenous solution. Baghdadite nanoparticles were added and mixed to obtain a homogenous suspension.

Table 1 shows the designation of the samples with PCL matrix in various amounts of baghdadite nanoparticles. The suspension was ultrasonically treated for 30 min before casting to achieve a more uniform dispersion and remove bubbles. The suspension was poured into the metallic mould and dried at room temperature for 24 h. After complete drying, the films were detached from the mould.

### 2.4. Morphological Characterisation of PCL-Baghdadite Films

Transition electron microscopy (TEM) (Philips EM208S, operating voltage 100 kV, Amsterdam, The Netherlands) is used to determine the particle size and perform the morphological studies of baghdadite powder. The sample’s surface morphology was observed using scanning electron microscopy (SEM, Seron AIS 2100, Philips XL30, 30 kV acceleration voltage, Uiwang, Republic of Korea).

The X-ray diffraction (XRD) was carried out using a Philips X’Pert MPD diffractometer. BAGHDADITE’s nano crystallite size (*D*) was obtained using the modified Scherrer equation in Equations (1) and (2) [41].

*L* = *Kλ*/(*β* cos *θ*) = (*Kλ*/*β*) (1/cos *θ*) (1)

*Ln L* = *Ln*(*Kλ*/*β*) + *Ln*(1/cos *θ*) (2)

where *K* is a constant related to crystallite shape, generally considered to be 0.9, *β* is the peak width of the diffraction peak profile at half the maximum height achieved from small crystallite size, and *λ* is the X-ray wavelength in nanometres. The value of the *β* in the 2θ axis of the diffraction profile is in radians [41].

### 2.5. Mechanical Characterisation and Modeling

The samples were prepared based on ASTM D 882 to characterise the tensile properties of thin plastic films [42]. The tensile test was performed on a Testometric Hounsfield H25KS universal testing machine (10 mm/min crosshead speed). The test specimen’s dimensions were 0.08 × 10 × 50 mm. The standard deviations and average values were ascertained from testing three samples of any nanocomposite system.

Also, the experimentally obtained mechanical properties of the fabricated composites are compared using the models of Chow [43], Ponte Castaneda [44], Counto [45], and the Generalised self-consistent scheme (Gscs) [46], Halpin Tsi [47] and Nielsen [48]. These analytical, semi-empirical models predict the elastic modulus of composite systems in which filler particles modify the polymer matrix. In selected models, the particles are assumed to be uniform in size and firmly bonded to the matrix. Both the filler and matrix are homogeneous and isotropic, including the interaction of filler with filler. Factors such as the volume fraction of particles, dispersed-phase Poisson’s ratio, Young’s modulus of particles, and Young’s modulus of the matrix influence the relative Young’s modulus of the composite.

### 2.6. Thermal Study

Thermogravimetric analysis (TGA) was used to study the thermal degradation of the fabricated baghdadite/PCL nanocomposites using a TA Instruments STA (Bahr 503 system analyser, Munich, Germany). Specimens of roughly 10 mg were placed in alumina containers and tested at a heating rate of 10 °C/min in a vacuum atmosphere with a thermal gradient over a temperature confine of 25 to 600 °C. The thermal behaviour of the baghdadite/PCL nanocomposites was studied using the differential scanning calorimetry (DSC) device (Sanaf, Tehran, Iran) in a nitrogen atmosphere. The samples were heated from −80 to 80 °C at a heating rate of 10 °C/min. The melting parameters were obtained from the heating scans. In order to characterise the crystallinity of PCL and composite film behaviour, Equation (3) was utilised, where $X_{cr}$ is the degree of crystallinity, $H_{f}$ is the heat of fusion obtained in experimental conditions, $W_{p}$ is the polymer weight fraction in the composite system, and $H_{f100}$ is the heat of fusion for 100% crystalline polymer (for PCL, it was considered 136 J/g [49]).

(3) $$X_{cr}\left(\%\right)=\frac{H_{f}}{W_{p}\times H_{f_{100}}}\times 100$$

### 2.7. In Vitro Bioactivity Evolution of Nanocomposite Films

The amount of bioactivity and, more precisely, the ability to form hydroxyapatite on the surface of the nanocomposite structure were investigated by placing it in a simulated body solution (SBF). The simulated body solution was prepared by the Boehner method [50]. PCL films consisting of various amounts of baghdadite nanopowder (n = 4) with a 10 mm × 10 mm dimension were immersed in SBF solution (pH 7.4) at 37 °C for four weeks. The pH value of SBF solutions containing samples was measured through the soaking time, utilising a pH meter (Metrohm, Herisau, Switzerland). Furthermore, EDS analyses and SEM were used to examine the apatite-formation capacity on the sample’s surface.

### 2.8. Ion Release Study

The samples’ calcium, silicon, and phosphorous ion release profiles were recorded using Inductively Coupled Plasma Mass Spectrometry (ICP-OS, Varian, ES 730, Palo Alto, CA, USA) for 28 days. Nanocomposite samples that were 300 microns thick were immersed in the Simulated Body Fluid (SBF) at 37 °C. Each time, the ion concentrations were assessed related to the amount of fresh medium, and the collective concentration of released ions was reported over 28 days.

### 2.9. Degradation Test

The mass loss method was used according to ASTM F1635 to measure the degradation rate of nanocomposites [51]. In this method, the films of 10 × 10 mm^2^ were first weighed (3 pieces for each composition). After this stage, the samples were placed in phosphate-buffered saline solution (pH = 7.4) at a steady temperature of 37 °C. The films were set in phosphate-buffered saline (PBS) solution for 7, 14, 21, and 28 days. The washed samples were then placed in the desiccator until the specimens were dried out entirely and weighed again. The destruction rate of samples (percentage of degradability) was calculated using the following equation:

% *Mass loss* = [(*W*_0_ − *W_t_*)/*W*_0_] × 100 (4)

where *W*_0_ is the original initial weight while *W_t_* is the weight at time *t* after drying (after soaking in PBS). The alterations in the surface morphology of samples were investigated utilising SEM images after the degradation period.

### 2.10. Biocompatibility of Nanocomposite Films

The cytotoxicity of PCL-based films was investigated by an indirect 3-(4,5-dimethylthiazol-2-yl)-2,5-diphenyltetra-zolium-bromide (MTT, Sigma, Saint Louis, MO, USA) assay. To ensure the removal of solvents and disinfect any contamination toward in vitro cell experiments, all films were washed with 70% volume ethanol in water and then dried. MG63 cells (human osteoblast cells purchased from the Royan Institute, Teheran, Iran) were used as favourable cell types for investigating the biocompatibility of the nanocomposite films with osteoblasts. A total of 10^4^ cells/mL were seeded on the PCL-based composites in 96-well plates for 24 h using Eagle’s medium Alpha modified supplemented with 10% fetal bovine serum (FBS) and 1% streptomycin/penicillin under standard culture conditions (5% CO_2_ and 100% humidity, 37 °C). The media was refreshed every two days. After removing the medium on days 1, 3, and 5, 100 μL MTT agent was inoculated into each well and incubated for 4 h. Next, 100 μL DMSO was added to the well to dissolve the formazan crystals, and the ELISA Reader (Stat Fax-2100, Miami, FL, USA) was used for detecting absorbance (at 545 nm).

The MG63 cells viability was calculated using Equation (5), where A_s_ is the absorbance of the treated sample, and A_b_ and A_c_ are the blank (DMSO) and control (tissue culture plate) (TCP) absorbances, respectively.

(5) $$\%\;\mathrm{relative}\;\mathrm{cell}\;\mathrm{viability}=[(A_{s}-A_{b})/(Ac_{\;}-A_{b})]\times 100$$

Also, the morphology of MG63 cells on PCL-based composite films was investigated using SEM images. A DAPI staining of living MG63 cell nucleus (4′,6-diamidino-2-phenylindole, blue fluorescence in live cells) in contact with PCL and PCL-based composite films was used to examine the proliferation of the cells. After washing with PBS, MG63 cells were fixed in 10% paraformaldehyde (Sigma Aldrich, Darmstadt, Germany) for 20 min, stained with DAPI, and photographed using a fluorescence microscope (Nikon TE 2000-U, Tokyo, Japan).

### 2.11. Statical Analysis

GraphPad Prism Software evaluated statistical analysis (V.6). Every experiment was performed at least three times, and the mean and standard deviation of the results were published (SD). Differences were declared significant at a probability error (p) of *p* < 0.05.

## 3. Results and Discussion

### 3.1. Characterisation of Baghdadite Powder

**Figure 1:**
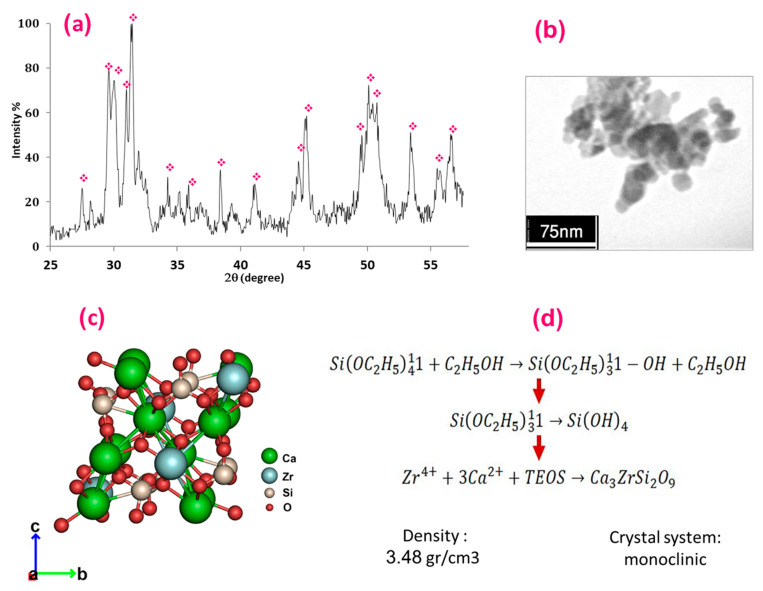
(**a**) XRD pattern of nano-baghdadite, (**b**) TEM image, (**c**) crystal structure [52], and (**d**) the formation mechanism of synthesised baghdadite powder.

The XRD pattern of synthesised baghdadite powder is presented in Figure 1a. The XRD pattern wholly matched with the standard card of baghdadite (JCPDS 00-016-0155), confirming the presence and synthesis of pure baghdadite with no impurities. According to the modified Scherrer equation, the crystallite size of baghdadite was measured at around 25 nm.

Figure 1b presents the TEM image of the synthesised baghdadite powder. As illustrated, the particles display a distinct spherical shape, emphasising the successful fabrication process. The size of these baghdadite particles was found to be within the 20–70 nm range, confirming their classification as nanoparticles. Furthermore, the mean particle size of the baghdadite was determined to be approximately 30 nm. Similar grain sizes for baghdadite crystals have been reported in previous findings [39]. Particle agglomeration is noticeable alongside the relatively uniform distribution of baghdadite within the matrix. This agglomeration signifies the high surface energy inherent in nanoparticles and their propensity to form clusters, corroborating earlier reports [53,54].

Baghdadite (Ca_3_ZrSi_2_O_9_) features a monoclinic crystal structure, as depicted in Figure 1c, which is a component of the CaO–ZrO_2_–SiO_2_ system. Baghdadite is categorised under the cuspidine group, a collection of silicates with a typical formula M_4_(Si_2_O_7_)X_2_. Here, M represents a cation of variable charge and ionic radii that typically occupy an octahedral space, while X can be OH, F, or O. As depicted in Figure 1c, baghdadite presents ZrO_6_ with six Zr–O distances that vary from 1.97 Å to 2.2 Å [55]. Figure 1d depicts the process of baghdadite formation. The initial step involves the hydrolysis of TEOS (reaction 1). Provided a sufficient amount of ethanol (H^+^) and a catalyst, such as HNO_3_, are used in this study, complete hydrolysis of TEOS can occur, as outlined in reaction 2. Before large silica molecules form a polymeric network in the solution through a condensation process, Zr^4+^ and Ca^2+^ are introduced to the solution to prevent segregation. Following these steps, the next phase of baghdadite synthesis can be seen as reaction 3 in Figure 1d. Various orthosilicates with different unit cells and symmetry patterns can connect with these octahedral walls. A second distinguishing feature of these minerals is the ionic distribution within the polyhedron, which is tied to their crystal chemistry [55].

### 3.2. Morphological Properties

**Figure 2:**
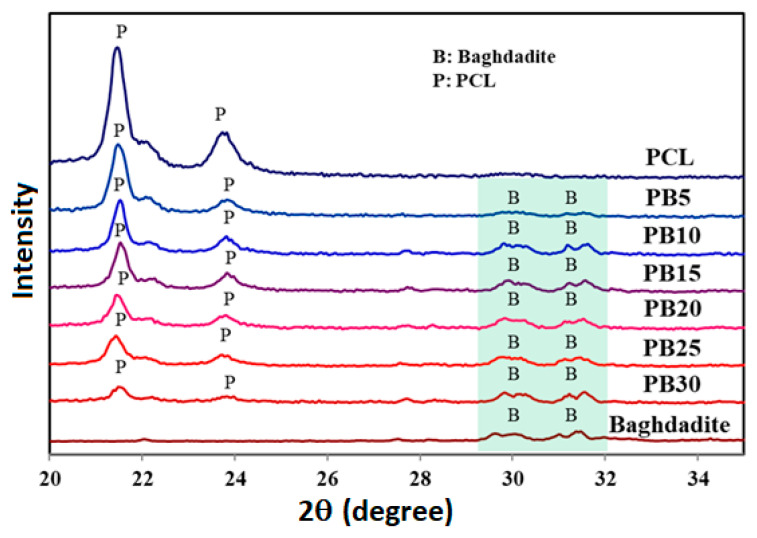
XRD patterns of PCL, PB5, PB10, PB15, PB20, PB25, and PB30. The change in colour from top to bottom indicates an increase in baghdadite.

The XRD patterns of the baghdadite-reinforced nanocomposites at different nanopowder loadings are shown in Figure 2. The XRD pattern in the case of pure PCL shows the presence of a semi-crystalline structure due to the existence of hydrogen bonds of the hydroxyl groups [56]. The XRD peak intensities of PCL demonstrate the main and highest peaks of PCL occurring between 20 < 2Ɵ < 25°, while the prominent baghdadite peaks are located at 29 < 2Ɵ < 33°. Although these two patterns do not overlap prominent constituent peaks, the increase in baghdadite wt.% exhibits an opposite effect on PCL’s peak intensity since PCL’s characteristic peak moves toward the lower angles.

Moreover, the results in the case of composite films indicate that adding baghdadite nanoparticles into PCL leads to a decline in the size of crystallites, as seen in Figure 2. In this regard, De Menezes et al. [57] reported a similar behaviour. The results, additionally, exhibit that pure PCL and PB30 lead to the most significant and lowest size of crystals, respectively, of all other nanocomposites. The findings could be connected to the presence of filler inclusions among the polymer chains acting as nucleating sites for crystal formation. This result is consistent with Khan et al.’s research [58] on the effect of particles on polymer chain crystallinity. The observations could also be considered as the high evaporation rate of the solvent. This acts as a barrier against the growth of crystallites in the PCL matrix. Polycaprolactone’s decrease in crystal peak intensity demonstrates that baghdadite particles are distributed and dispersed in the polymer matrix.

**Figure 3:**
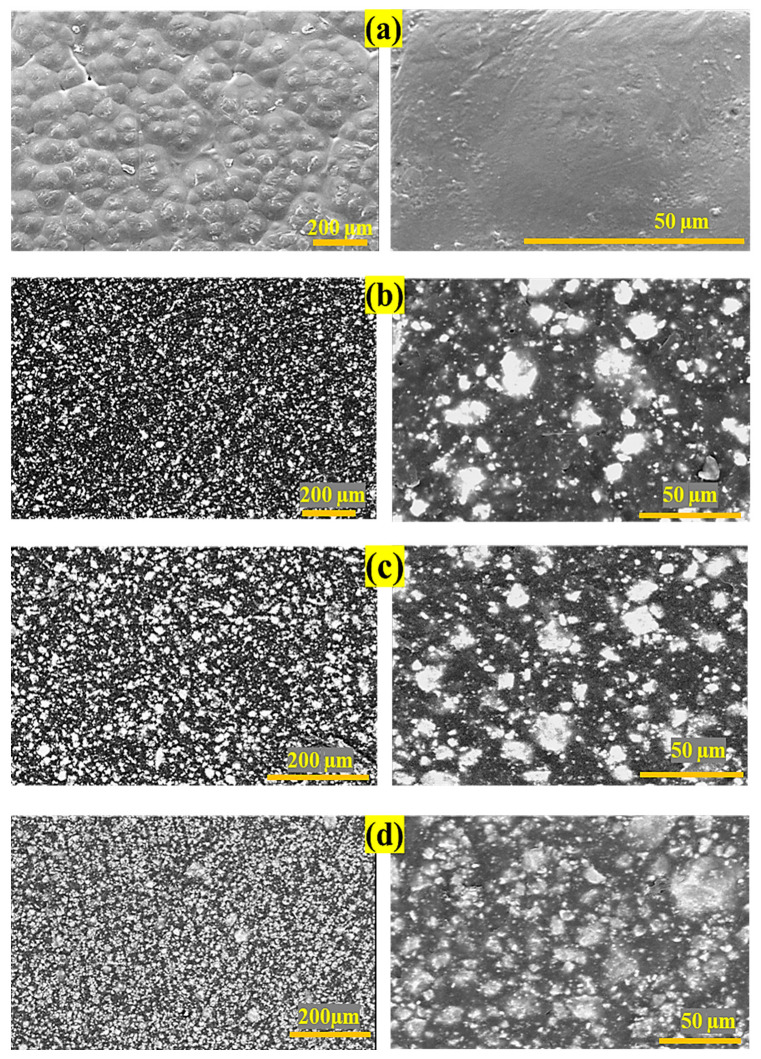
SEM images of baghdadite/PCL nanocomposites. (**a**) PCL, (**b**) PB10, (**c**) PB20, and (**d**) PB30.

The SEM images were used to determine the dispersion and distribution of fillers within the matrix after processing. Figure 3 represents the surface morphology of the pure PCL and those of nanocomposites reinforced with 10 to 30 wt.% of baghdadite. The images show that the fabrication process yields an appropriate relative level of dispersion, as represented by the baghdadite powder. The increase in the amount of baghdadite in the parent polymer does not significantly alter the distribution level of fillers in the matrix. However, with the rise of reinforcing particles in the composite, agglomeration increased too [59].

### 3.3. Thermal Properties

**Figure 4:**
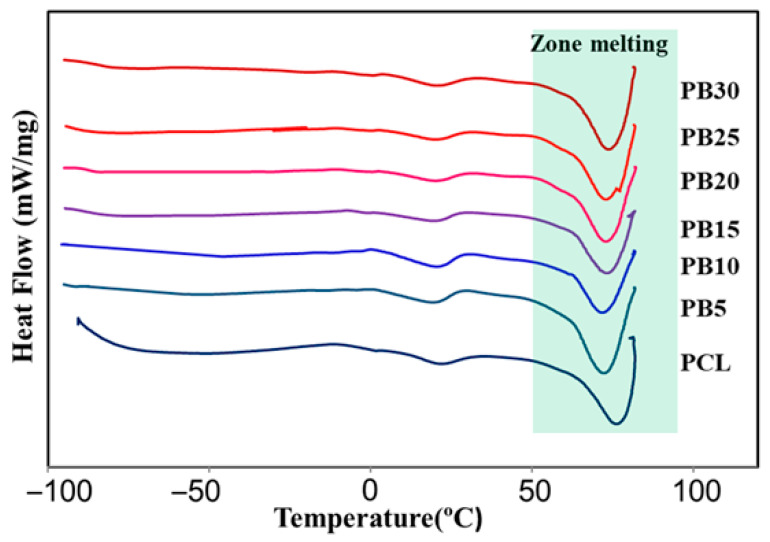
DSC thermographs of PCL, PB5, PB10, PB15, PB20, PB25, and PB30. The change in colour from top to bottom indicates the decrease in baghdadite.

The thermal characteristics of the specimens were assessed to determine better key interplays between the structure and the eventual mechanical response of the samples. Figure 4 shows the DSC traces obtained by thermal characterisation of 0–30 wt.% baghdadite reinforced PCL. The DSC analysis results reveal that the melting temperature of the PCL specimen decreases from 76.1 °C to 72.3 °C and 71.6 °C when 5% and 10% of baghdadite are used. The results also indicate that the melting temperature of the nanocomposites remains invariant and reaches ~73.4 °C by adding a higher wt.% of baghdadite. 

The observations could be correlated to the presence of competitive effects that independently contribute to the melting point of composites. First, adding baghdadite nanoparticles to PCL could decrease crystal thickness since the particles provide a more significant number of nucleating sites, as described earlier. It has been broadly reported that thinner crystallites result in lower melting temperatures. Second, the greater density of the added filler at higher loading produces more pinning sites, which impede crystal growth in composites loaded with a high wt.% of baghdadite. This phenomenon influences both primary nucleation rate and crystal/spherulite growth.

Table 2 shows the degree of crystallinity of PCL and composite films. In general, with the addition of baghdadite, a decrease in the degree of crystallinity of the composites is observed at first. The crystallinity degree, Xcr, decreased when fillers were added to composites (the most significant decrease in the sample containing 10 wt.% Baghdadite). In nanocomposite films, crystallisation behaviour may be decreased due to reduced mobility of the polymer chains caused by fast nucleation on the surface of the filler.

However, with the increase in the baghdadite’s weight percentage, the crystallinity level increases again and remains almost constant in the same range. In contrast, the increase in filler content (15 wt.% and more) made this effect less prominent. Heterogeneous nucleation on the surfaces of the fillers may be the primary process behind the matrix’s crystallisation behaviour in polymer/filler composites [60]. Therefore, depending on the nucleation abilities and baghdadite wt.%, the values of Xcr may be increased or diminished in different content of baghdadite-PCL composites. Similarly, the effect of filler addition on the crystallinity properties of polymer composites was investigated by other researchers [61,62,63]. The nucleation is hampered due to increasing the filler content, as previously indicated, and the values of Xcr closer to levels of the neat PCL. This outcome is explained by the ability of the filler’s more significant volume fraction to counteract agglomeration at higher filler contents.

**Figure 5:**
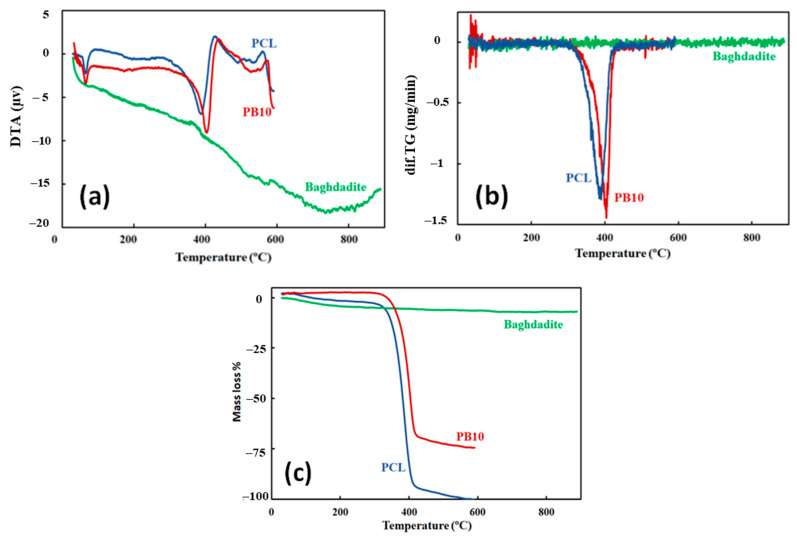
(**a**) DTA, (**b**) derivative of TG, and (**c**) TG curves of PCL, baghdadite, and PB10 as a function of temperature.

Representative DTA tests were performed to understand better specimens’ thermal behaviour concerning changes in the composites structure. Figure 5a indicates that adding 10 wt.% baghdadite to the neat PCL increases the degradation temperature from 388 °C to 402 °C. Likewise, the TG test showed the same results (Figure 5b), confirming the increase in degradation temperature. The findings corroborate the hypothesis that baghdadite/PCL composites exhibit a more significant thermal damage threshold than pure PCL by enhancing the interaction at the interface of baghdadite and PCL. It can be concluded that baghdadite contributes relatively to improving the thermal stability of PCL nanocomposite films.

Representative TGA analyses of specimens during the heating scan are shown in Figure 5c. This Figure illustrates the mass loss of the PCL and baghdadite phases compared with that observed in composites with 10 wt.% baghdadite reinforced PCL. The results reveal that all the mass of pure PCL is decomposed when the temperature reaches 600 °C. In contrast, the thermal stability of 10 wt.% PCL nanocomposites has improved compared with that of pure PCL. The outcomes are consistent with the findings of Abdolmohammadi et al. [64], where adding calcium carbonate to PCL improved its thermal and mechanical properties. The mass loss traces further show the reduction in mass loss of specimens reinforced with 10 wt.% baghdadite and indicate that 75% of the mass is evaporated during the thermal scan of specimens from room temperature to 600 °C. This temperature variation may be attributed to a transition from the simple chain scission degradation process that is typical of raw PCL [65] to a more complicated, two-step nucleation-driven degradation process, which implies the degradation process begins at discrete points (in the presence of baghdadite) and then spreads to the remaining polymer [66]. The TGA representative curves also signify a mass loss of ~7% in the case of pure baghdadite at the end of the heating scan. The results obtained can be attributed to the presence of moisture in baghdadite samples, which cannot be avoided due to the time scale from sample preparation for testing.

### 3.4. Mechanical Properties

**Figure 6:**
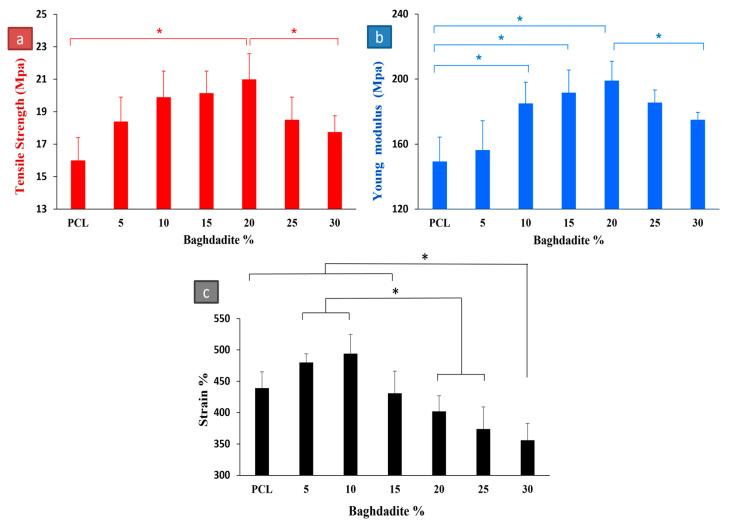
(**a**) Tensile strength, (**b**) Young’s modulus, and (**c**) strain rate of Baghdadite/PCL nanocomposites films. Significant differences between the samples are shown by “*” (*p* < 0.05).

The tensile strengths, Young’s modulus, and strain rate of the pure PCL film and the PCL-baghdadite nanocomposite films are represented in Figure 6. It is clearly shown that pure PCL attains the lowest tensile stress strength among all fabricated composites. In contrast, the PB20 yields the most significant strength, around 20 MPa. Moreover, as shown in Figure 6, the elongation values of films decrease by adding baghdadite. Particularly after PB10, the strain rate of all samples is under 450%.

**Figure 7:**
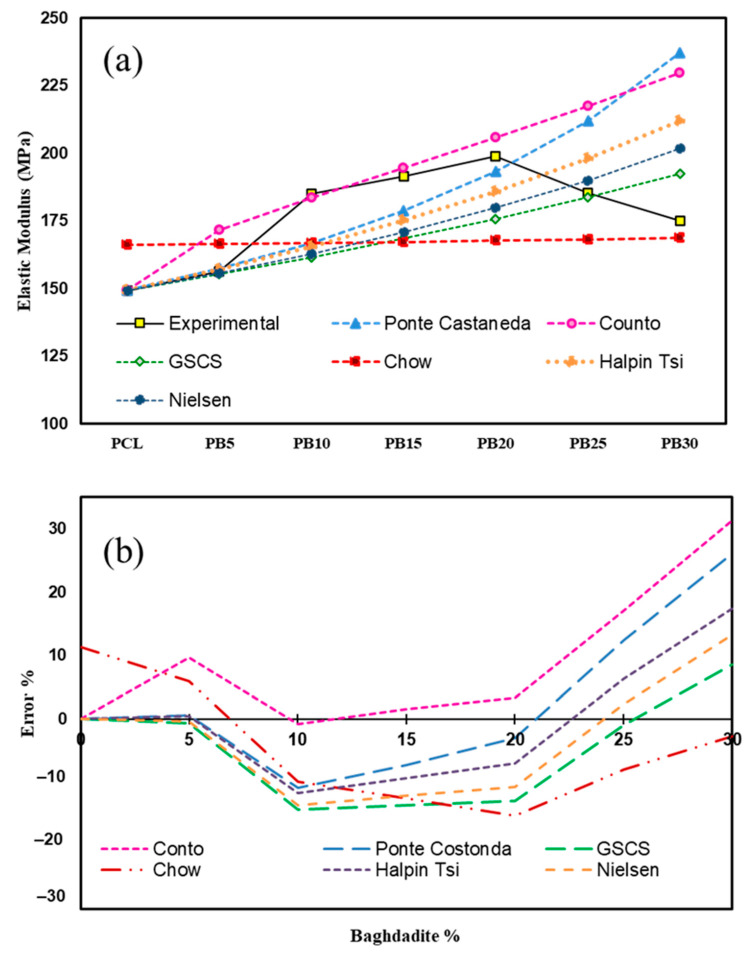
(**a**) Elastic modulus of experimental results and prediction models of PCL, PB5, PB10, PB15, PB20, PB25, and PB30. (**b**) Error percentages of prediction elastic modulus of selected models in different baghdadite wt.%.

Figure 7a shows the results of prediction models compared to the experimental data. Figure 7b indicates the error percentages of the models based on baghdadite wt.%. The highest and lowest error percentages in the prediction of the elastic modulus of composite in Conto are 31.29 and 0.76, in Ponte Castaneda are 26.15 and 0.65, in Chow are 15.21 and 2.74, and in GSCS are 14.34 and 0.68 Mpa.

In the case of reinforced composites, the matrix mostly bonds the loading. Particle dispersion in the matrix impedes molecular chain movement [67]. This explains why the PCL composite containing baghdadite was of higher strength than the pure PCL. The modulus decreases as the filler is added due to large agglomerates acting as cracking initiation sites as the filler amount increases. The modulus decreases as the filler is added due to large agglomerates acting as cracking initiation sites as the filler amount increases [68]. The results are consistent with a study published on HA as a filler in polymer composites [69]. A poorly dispersed matrix may result in the agglomeration of HA particles in the composite, resulting in poor strength properties [70].

The results show that Ponte Castaneda has proper adaptability up to 20% baghdadite, where the error rate is less than 10%. In PB25 and PB30, the error slope increases as the error increases to 25% and 30%, respectively. Therefore, this model can provide a suitable estimate of the elastic modulus up to 20 wt.% baghdadite particles in polycaprolactone. Formerly, Bergstrom et al. [71] reported that this model predicted the experimental data up to 25% of the filler volume fraction.

Chow’s model has a very conservative estimate of the effect of increasing the percentage of baghdadite on the increase in strength of the composite. Its elastic modulus is best estimated at 30% baghdadite wt.% because of this reason. While in Counto and Ponte Castaneda’s models after PB20, the elastic modulus increases significantly and causes more error in the estimates, this model has a more accurate estimate.

Counto is the best model until 20% of the filler. The error of this model in this interval is less than 5%. Halpin Tsi and Nielsen have similar behaviour in estimating the strength value, with the difference that Nielsen always predicts more conservative numbers of elastic modulus. At 25% by weight of baghdadite, four models (Halpin Tsi, Nielsen, CGSC, and Chow) predict the elastic modulus of the composites with an error of less than 10%. The generalised self-consistent scheme shows the most accurate prediction in PB25. Also, based on the results, the two GSCS and Chow models with a high weight percentage (30) have proper accuracy with less than 10% error. Mainly, GSCS has fewer approximations than the four models.

As shown in Figure 6, adding the baghdadite particles enhances the mechanical properties as theoretically expected [72]. The observations suggest that a proper interfacial interaction exists at the baghdadite nanoparticles and polymer interface and an appropriate level of baghdadite dispersion [73]. The results thus signify quite good bonding at the interface of the baghdadite polymer, corroborating that the reinforcement phase shares its mechanical properties with the parent PCL matrix. Moreover, it is clearly shown that the tensile strength and elastic modulus concurrently decrease at the more significant loadings of the reinforcements. This result was also obtained by Liang et al. [74]. This finding could be attributed to various mechanisms available due to the filler–filler and filler–polymer interactions.

The agglomeration of baghdadite particles decreases the surface-to-volume ratio (specific volume) and the available surface area between the reinforcements and polymer. Thus, a reduction in the level of interfacial load transfer [17]. Moreover, as explained earlier, it has been frequently shown that fillers might act as nucleating agents in the formation of crystals in semi-crystalline polymers. The mechanical properties that are reduced when more remarkable filler contents are used could be attributed to the amount and size of the crystals created in the matrix [75]. The other reason worth mentioning is the slippage sites among the reinforcements when the surface of the particles has not been well-wetted by the polymer phase resulting in an agglomerated phase and, consequently, the existence of interfacial voids and micro-crack sites. Previous studies have widely reported that the agglomeration phase cannot be effortlessly avoided during the nanocomposites’ fabrication process, particularly at more remarkable filler contents. The extensive surface area of the reinforcement phase at the nano-size level is one main factor leading to the poor dispersion of nanoscale materials in a polymer matrix [76,77].

Since the presented models do not consider cases such as particle size, particle interaction, and the distribution of particles in matrixes. Further, with the increase in weight percentage, due to the high surface energy of the nanoparticles, they become agglomerated, their level of uniformity in the field decreases, the probability of creating bubbles in the sample increases, and the amount of interaction between the particle and the matrix decreases. As a result, the mechanical properties reduce, so in the theoretical models, ideally considering conditions, an increase in weight percentage leads to more volume occupied by the filler material and, finally, an increase in mechanical properties in the ideal situation.

### 3.5. Bioactivity Assessment

**Figure 8:**
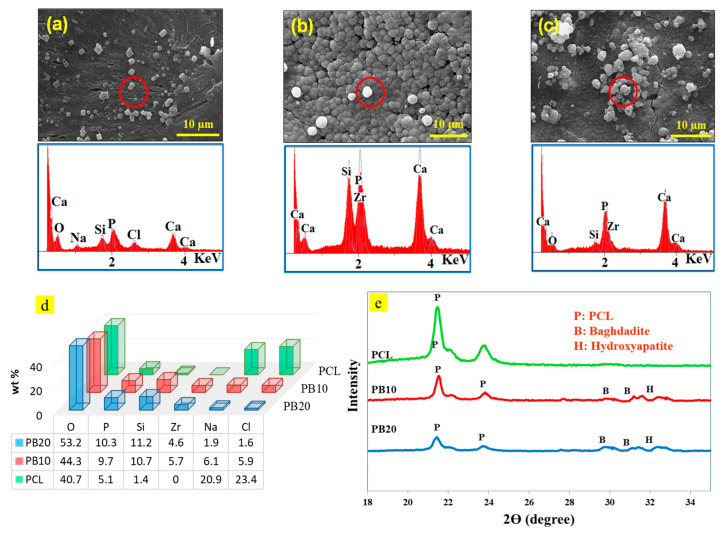
SEM images and EDS of samples after 28 days of soaking in SBF solution on the surface of (**a**) PCL, (**b**) PB10, and (**c**) PB20. (**d**) EDS analysis of composite films after 28 days of immersion in the SBF solution. (Percentage distribution of elements in PCL, PB10, and PB20). (**e**) XRD patterns of composite films after 28 days of immersion in the SBF solution.

Three compounds were selected for bioactivity evaluation. Figure 8a–d show the SEM images, XRD patterns, and energy dispersive X-ray analysis (EDS) of PCL, PB10, and PB20 after 28 days of immersion in the simulated body solution (SBF), respectively.

While no apatite of the angular crystal shape was formed on the PCL film, white particles were observed on the pure PCL surface. Their morphology is entirely different from the spherical morphology of hydroxyapatite particles. These particles are the precipitation of salt crystals from the simulated body solution compounds; therefore, the PCL film does not have much bone growth potential. Formerly, similar behaviour was noted for many polymers and polycaprolactone [78,79,80]. The images show the growth of hydroxyapatite particles on the film surfaces containing baghdadite particles. However, the PB20 has a higher apatite density, and the accumulation of white apatite precipitation can be seen on the sample’s surface, suggesting a high degree of bioactivity.

As seen in Figure 8d, the precipitation on the PCL film’s surface is rich in sodium and chlorine, resulting from the body’s simulated solution. Sediments formed on composite films containing baghdadite nanoparticles contain calcium and phosphorus. The calcium-to-phosphorus atomic ratio is approximately 1.74 (PB10) and 1.67 (PB20). These atomic ratios of calcium are very close to the phosphorus of apatite (1.67) [81], which exhibits the apatite formation on the surfaces of the composite film. Therefore, it could be concluded that raising the number of nanoparticles could increase the bioactivity of the specimens.

XRD pattern of specimens (Figure 8e) indicated sharp peaks at 2Ɵ = 31° belonged to hydroxyapatite, which confirmed the bioactivity of PCL-based films containing baghdadite particles. Similar results, reported by Soleymani et al. [82], confirmed the bioactivity of baghdadite nanoparticles. They developed chitosan/PCL baghdadite nanocomposite as a bioactive coating for magnesium alloys [82].

### 3.6. Ion Release Study and pH Evaluation

**Figure 9:**
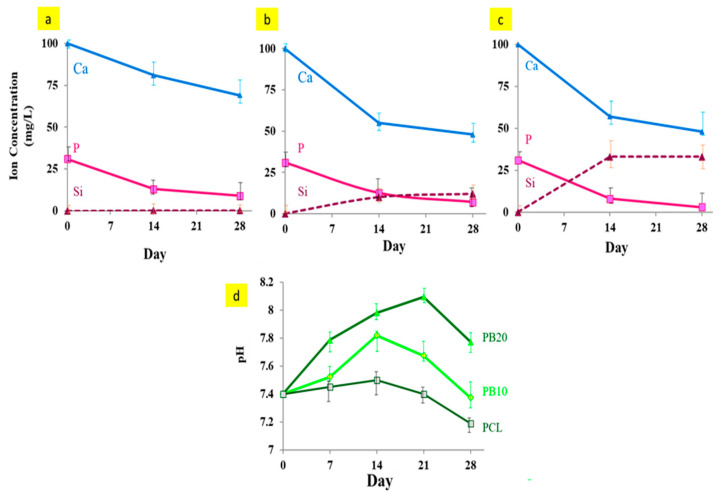
Changes in the concentration of Ca, P, and Si ions in SBF solution during 28 days of immersion of films including (**a**) PCL, (**b**) PB10, and (**c**) PB20. (**d**) XRD patterns of composite films after 28 days of immersion in the SBF solution.

Figure 9a–c show the trend of variations in the calcium, phosphorus, and silicon ion concentrations after 28 days in the simulated body solution. These curves are plotted from the inductively coupled plasma mass spectrometry (ICP) test results. According to studies [83,84], silicon release in the baghdadite compound due to ion transfer between the baghdadite nanoparticles and the ions in the simulated body solution is the principal bioactive factor of baghdadite films. As a result of this displacement, the silicate bonds of baghdadite (Si-O-Si) are broken, and the Si-OH bonds (hydrophilic groups of silanol) are formed on the surface of the nanoparticles. The ion concentration variations in the simulated body solution after 28 days of immersion (Figure 9a–c) also release silicon ions and reduce the percentage of calcium and phosphorus ions. These results confirm that the apatite phase deposition is present on the surface.

By releasing silicon ions from baghdadite nanoparticles into the SBF and forming silanol groups, suitable places are provided for phosphorus and calcium ion deposition from the simulated body solution [85]. Accordingly, the increase in the bioactivity of composite films using baghdadite nanoparticles is due to their partial dissolution and release of silicon ions, which provide a suitable surface place on the films for apatite formation. In addition, it seems that the separation of baghdadite nanoparticles exposed to the SBF increases the surface roughness of the film and provides suitable germination sites for the apatite phase to grow.

With a further increase in baghdadite nanoparticles in composite samples, the amount of calcium absorption from the SBF and the formation of more apatite on the surface of the films increases, so the concentration of this ion, according to Figure 9a–c, gradually decreases in the simulated body solution. Figure 9d shows the pH changes of the SBF solution at different immersion times in films containing different amounts of baghdadite nanoparticles. The pH of the SBF initially increases over a 28-day period, which is 7.8 and 8.1 for PB10 and PB20, respectively. Subsequently, these values decrease and reach 7.3 and 7.7 at the end of 28 days. In the case of pure PCL, the pH decreases slowly to about 7.2. The reduction in pH is because of the slight degradation of the polymer chains and the release of small polymer chains with acidic ends, as mentioned in other research [86,87].

The acidic products due to the degradation of PCL enters the solution and leads to a slight decline in the pH of the solution. During the first weeks, the dissolution of baghdadite particles leads to replacing silicon and calcium ions with H^+^ ions from the SBF solution, increasing the pH. Then, with the deposition of calcium and phosphorus on the films and their concentration in the solution, the alkaline environment decreases, and the pH decreases again. In fact, within the first weeks, particle dissolution is predominant. In the end, sedimentation and apatite formation are the leading causes of pH changes.

### 3.7. Degradability Assessment

**Figure 10:**
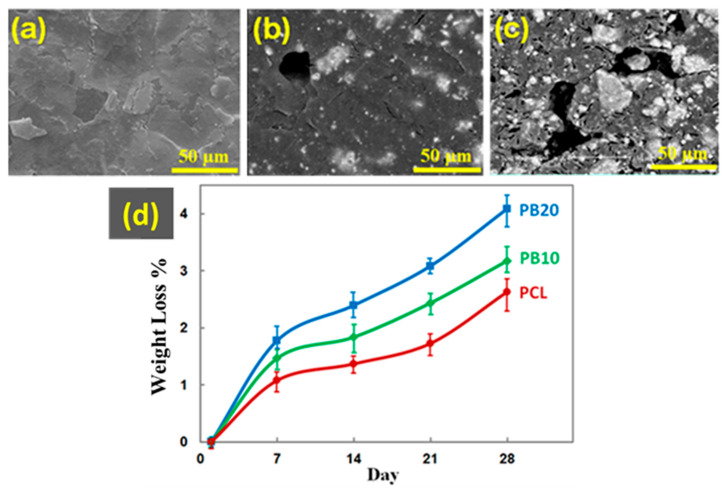
SEM images of (**a**) PCL, (**b**) PB10, and (**c**) PB20 after 28 days of immersion in the PBS solution. (**d**) Graph of the weight loss percentage of different composite films as a function of baghdadite wt.%.

Figure 10 shows scanning electron microscopic images and the degradability of composite films after 28 days of immersion in PBS solution, respectively.

As seen in the images, the degree of degradability grows by increasing the baghdadite content. Most of the rupture points are located in the vicinity of baghdadite particles. The findings reveal an increase in the rate of degradation of fabricated films in the presence of this bioceramic in PBS solution. It was previously noted that PCL has a low rate of degradation and bioactivity, which could be modified by composite construction to improve its biological properties, as mentioned in previous studies [25]. As can be seen from Figure 10, the increase in baghdadite nanopowder content has led to the further weight loss of the composite films. After about one week of immersion of the samples in the phosphate-buffered solution, the weight loss rate of the samples becomes almost constant. Over time, the weight loss of all of the films also increases, so that at the end of day twenty-eighth, the weight loss of PCL, PB10, and PB20 is 2.63 ± 1.3%, 3.18 ± 1.5%, and 4.08 ± 1.69%, respectively.

The separation of silicon ions due to the nanometre-sized baghdadite in the construction causes the hydrolysis of Si-O-Si groups on the surface. It forms hydrophilic (Si-OH) groups, separating silicon and breaking the bonds containing this particle. Due to the interfacial interaction between ceramic nanoparticles and the polymer matrix, the presence of these groups at the interface increases the two components of film degradation. Furthermore, the increase in the degradation rate of the film caused by adding ceramic nanoparticles could be attributed to the increased reaction surface of the solution. In addition, it could enhance samples’ surface roughness [88,89].

### 3.8. Biocompatibility of PCL-Based Composite Films

**Figure 11:**
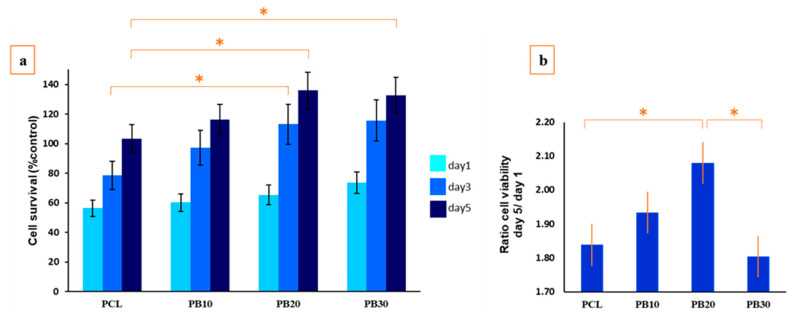
(**a**) Cell survival of MG63 cells cultured on the nanocomposite films and (**b**) cell viability ratio of the nanocomposite films on day 5/day 1. Significant differences between the samples are shown by “*” (*p* < 0.05).

**Figure 12:**
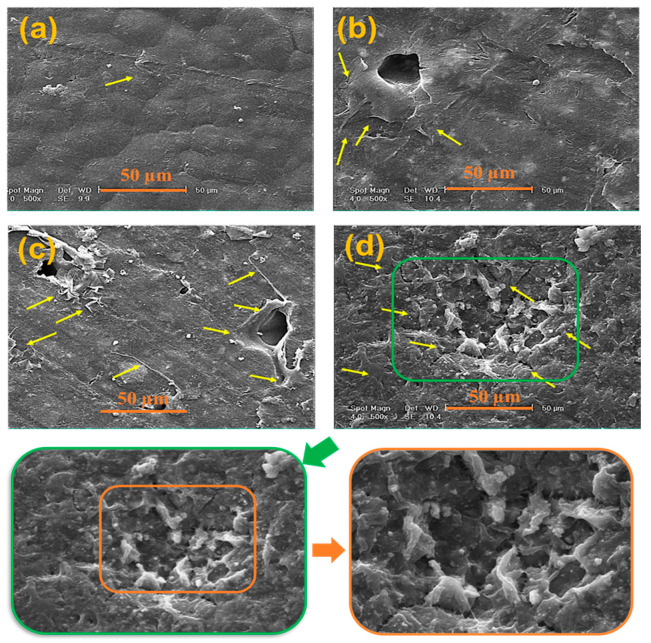
SEM image of MG63 cells cultured on (**a**) PCL, (**b**) PB10, (**c**) PB20, and (**d**) PB30 films. Yellow arrows mark some cells for easier identification.

MTT results for composite films after 1, 3, and 5 days are indicated in Figure 11a. MG63 cell viability ratios on PCL, PB10, PB20, and PB30 films are shown in Figure 11b. Also, for better confirmation of film cytocompatibility, SEM images are presented in Figure 12.

Figure 11 shows that cell growth increased regardless of the baghdadite percentages, increasing the culture time, which shows the biocompatibility of all fabricated films. After five days of cell culture, the number of living cells is almost twice as high as after one day of culture. As shown in Figure 12, some cells are marked for easier identification. The SEM images in Figure 12 revealed the excellent attachment of cells on the specimen surface, attributed to the Si, Ca, and Zr ions’ ability for bone cells to attach to the substrate. On the other hand, there is an apparent relationship between the extracted ions and cellular activity. Therefore, the presence of Si, Ca, and Zr ions in the culture media should be the main reason for better cell attachment, viability, and proliferation on PCL films containing baghdadite. Similar results were reported in the Pahlevanzadeh et al. study [85], which confirmed that incorporating baghdadite into PCL-containing substrates can enhance osteoblasts attachment and viability. The Si ions in this ceramic, including the silica group, may be negatively charged due to their lower isoelectric point, releasing the Si-OH groups in the media. The Si-OH groups could join functional groups containing growth factors, providing suitable sites on the Si-containing ceramics for cell growth [90].

Another effect on cell viability and adhesion is from porosity and film pores. Similarly, Wu et al. [91] reported that pores with suitable sizes could create desirable binding sites for cell attachment. Regarding the effects mentioned above, the sample with 20 wt.% of baghdadite exhibited optimal conditions for the sample’s biocompatibility. Also, in the Bedair et al. [92] study, poly(lactide-co-glycolide) (PLGA) composites containing magnesium hydroxide (MH) nanoparticles were fabricated via a solvent casting technique and modified with polydopamine (PDA) for morphogenetic protein-2 (BMP2) delivery. Their results demonstrated that a PLGA/MH scaffold could improve MC3T3-E1 cell proliferation and osteogenic differentiation, revealing ceramic-containing substrates’ appropriate cell behaviour [92]. In another study by Chahal et al. [93], better cell activity and particularly more mineralisation of extracellular matrix were observed for calcium phosphate-loaded poly(ethylene glycol) scaffolds, which can confirm the favourable effect of Ca-containing ceramics on bone regeneration.

Since PCL is a hydrophobic polymer with a very low degradation rate, utilisation of that as a coating cannot be a suitable suggestion. In this regard, in this study, opposite of Arefpour et al. [94], PCL is used as one part of nanocomposite films. Therefore, an improvement in cell behaviour is expected. On the other hand, the PCL coating can entrap baghdadite nanoparticles and prevent the release of favourable ions for bone regeneration from films, which is solved in this study.

**Figure 13:**
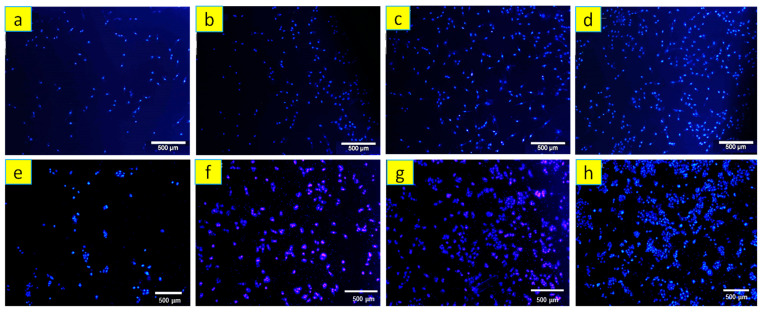
DAPI staining of MG63 cells on day 1 of culture: (**a**) PCL, (**b**) PB10, (**c**) PB20, and (**d**) PB30 films, and Day 5 of culture on composite films: (**e**) PCL, (**f**) PB10, (**g**) PB20, and (**h**) PB30.

The positive effect of baghdadite on higher viability and proliferation of both PB20 and PB30 is shown by DAPI staining of the MG63 cells cultured on the composite films in Figure 13. This effect may be attributed to baghdadite’s bioactivity and release of silicon and zirconium ions, which accelerates cell activity. The results show that adding Baghdadite to PCL creates a suitable substrate for the growth and promotion of MG63 cells in composite films. Also, with increasing cultivation time, cell growth and expansion in all samples were observed, resulting from the appropriate biocompatibility of composite films. But in PB20 and PB30, the cell growth and expansion rate are more impressive, which aligns with the viability test results.

A study by Ramaswamy Y et al. [95] explored how baghdadite affected human osteoblast-like cells, osteoclasts, and endothelial cells. Their findings are consistent with current results in that proper baghdadite cell interaction, where baghdadite, as compared to wollastonite, promoted cell proliferation and differentiation and facilitated the attachment of human osteoblast-like cells with a systematised cytoskeleton structure. Additionally, researchers have demonstrated that baghdadite has beneficial effects on the development and growth of cells [96].

## 4. Conclusions

This study focuses on developing biodegradable-bioactive nanocomposites using baghdadite nanoparticles as a reinforcement phase. The baghdadite was synthesised using the sol–gel method, and the PCL-based nanocomposites were fabricated with 0–30 wt.% baghdadite using the solvent-casting technique. The nano size of baghdadite particles was obtained from TEM images, and measuring the crystallite size of baghdadite powder was carried out with a modified Scherrer equation. The results of the tensile strength, the DSC, DTA, and TG characterisation showed that adding baghdadite to PCL improved the mechanical and thermal properties. Six modelling cases were considered to predict the composite system’s effective modulus. Analytical prediction models could give a good approximation of the mechanical properties.

Moreover, the baghdadite/PCL composites demonstrated enhanced bioactivity and apatite formation on the surface of composite films. The results of the degradability test showed that with the increase in baghdadite nanoparticles, the amount of decomposition of silicon-containing groups increased, and the rate of degradation of composite films increased compared to pure PCL films. The cell culture results indicated that the baghdadite particles positively affected cell growth, and the optimal composition contained 20 wt.% baghdadite, considering its mechanical properties. The findings also revealed that baghdadite could replace the existing bioactive fillers to fabricate biodegradable-bioactive bio-/nanocomposites with improved mechanical and thermal performance.

Overall, the study suggests that baghdadite is a promising alternative bioactive filler for fabricating biodegradable-bioactive nanocomposites with improved properties for bone regeneration applications.

## Figures and Tables

**Table 1:** Designation of PCL nanocomposites and their composition.

PCL (wt.%)	Baghdadite (wt.%)	The Abbreviated Name
100.00	0.00	PCL
95.00	5.00	PB5
90.00	10.00	PB10
85.00	15.00	PB15
80.00	20.00	PB20
75.00	25.00	PB25
70.00	30.00	PB30

**Table 2:** Crystallisation parameters of PCL and composite films (% of crystallisation).

PCL	PB5	PB10	PB15	PB20	PB25	PB30
81.9	72.4	51.6	59.6	67.6	67.2	64.6

## Data Availability

The data presented in this study are available on request from the corresponding author. The data is not publicly available because it is part of an ongoing study.
